# Semi-rigid ureteroscopy: indications, tips, and tricks

**DOI:** 10.1007/s00240-017-1025-7

**Published:** 2017-11-18

**Authors:** Lily A. Whitehurst, Bhaskar K. Somani

**Affiliations:** 1 Department of Urology, Royal Hampshire County Hospital, Romsey Road, Winchester, SO22 5DG UK; 2 Department of Urology, University Hospital Southampton NHS Foundation Trust, Southampton, SO16 6YD UK

**Keywords:** Urolithiasis, Semi-rigid ureteroscopy, Tips and tricks, Calculi, Ureterorenoscopy

## Abstract

Advances in ureteroscopic technology, alongside broadening treatment options have fuelled the rapid expansion of endourology. Semi-rigid ureteroscopy is a well-known procedure used globally for varying urological conditions, with high success rates. This article aims to provide ‘tips and tricks’ for the semi-rigid ureteroscopy procedure, and the management of commonly encountered pathology such as renal stones, ureteric strictures, and urothelial tumours.

## Introduction

Ureteroscopy (URS) was discovered opportunely in 1912 when a paediatric cystoscope was inadvertently inserted into the renal pelvis of a child with a dilated ureter; however, Young and McKay published these findings in 1929 [1]. The next development of the ureteroscope was the invention of the rod lens cylinder system by Hopkins in 1956, which allowed for narrower scope diameters and better light transmission, thus improving endoscopic access and image quality [2]. Further advances were limited by the slow adoption of fiberoptic technology into medical practice [1]. Finally, in 1980, urologist Perez-Castro, in association with Karl Storz, produced the first ureteroscope, which was a 12 F, 50 cm-long, rigid scope with a separate optic and working channel. The following year, it was successfully used for lithotripsy of a renal calculus [3]. The first semi-rigid ureteroscope was introduced in 1989, and it rapidly replaced the rigid model as it could allow flexion of up to 2 inches off the vertical axis without image distortion and, therefore, was less likely to fracture [2]. Technology has progressed tremendously since, with both flexible and semi-rigid ureteroscopes in wide usage globally [2–4].

Indications for URS include both diagnostic and therapeutic interventions for stone disease, strictures, and ureteric tumours in varying patient groups. Current European Association of Urology (EAU) guidelines state that both ureteroscopy and shock wave lithotripsy (SWL) have comparable stone free rates (SFR) for most ureteric stones. Whilst URS or SWL can be used for stones < 10 mm for any ureteric stone location, the EAU advocates the use of URS as first line for distal ureteric stones larger than 10 mm [5]. Complication rates for URS have improved, since their introduction and currently range from 9 to 25%, with majority of complications being managed conservatively [4, 5].

The aim of this paper is to summarize the current indications of semi-rigid ureteroscopy and provide “tips and tricks” for its successful undertaking.

## Pre-operative preparation and equipment

For all patients undergoing URS, there should be an adequate amount of planning. Correct imaging for the patient should be performed pre-operatively and should be displayed in the operating theatre for each procedure. Routine urinalysis should also be performed to exclude any evidence of infection. Informed consent should be gained from all individuals, and important risks of instrumentation such as failure to gain access to the ureter, ureteric perforation, and post URS strictures should be mentioned. A very small risk of ureteric avulsion (< 1%) and its consequences also need to be discussed [4, 5].

The World Health Organisation (WHO) style checklist should be performed for all cases with patient side marking and antibiotics as per local hospital policy. Each case should be set up in a standard manner; patients require positioning in the dorsal lithotomy position, ensuring that all pressure points are padded to prevent tissue and nerve damage [6, 7]. General anesthesia (GA) is recommended over spinal anesthesia if the patient is suitable, especially for prolonged procedures. GA also provides smaller tidal lung volumes, and therefore, breathing movement is less disruptive to the procedure, and mechanical ventilation can be temporarily stopped if required [8]. Fluoroscopy equipment should be available in the operating room (OR), and a warning radiation sign and/or a laser sign should be placed outside the OR [6, 7]. A suggestion for equipment set-up is with the radiology C-arm and endoscopic tower on contra-lateral sides, so that their placement does not interfere with each other [9]. Warmed normal saline is the standard fluid used for irrigation, and it is usually pressurised to allow adequate vision at the tip of the scope [2]; however, pressure in the renal system should not exceed 30 cm H_2_O as this can lead to post-operative pain and risk of intratubular backflow with fornix rupture [7].

Any additional equipment that may be required during the procedure should be prepared and be readily available before starting the case. This includes guidewires (both as a safety and working wire), which are important in gaining access and navigation into the renal system, and they also allow ureteral catheters, scopes, and stents to be repeatedly inserted with minimal trauma. There are varying types of guidewire that exist, but the important characteristics are a flexible tip, low friction, and a rigid shaft [6]. Guidewires tend to be coated with polytetrafluoroethylene (PTFE) or a hydrophilic polymer, which must be kept moist prior to its insertion as this facilitates placement and helps to protect the working channel of the scopes. Stone extraction devices are essential for the removal of fragments and tend to be made of nitinol, which is suited to stone work as the devices can maintain their shape and refrain from kinking [6]. Nitinol instruments tend to be thinner than other instruments, which means that they are less occlusive to the working channel and tend not to impede vision by reducing irrigant flow [10].

Stone fragmentation during rigid URS is achieved either via the pneumatic or a laser fragmentation device, although the latter is gaining more popularity [7–11]. A laser machine should be set up prior to commencing a procedure, and appropriate safety guidelines must be adhered to with its use, for the safety of both the patient and staff. Pneumatic system can be used effectively with good stone disintegration during rigid URS; however, stone retropulsion into the kidney is a common problem, occurring in 5–40% of cases [12]. Smaller stones, greater proximal ureteric dilatation, and significant hydronephrosis are risk factors for stone migration, and this leads to increased patient morbidity and cost [12]. This can be prevented by placement of special anti-retropulsion devices, such as ureteral occlusion devices often formed of nitinol wires; for example, the Stone Cone^™^ by Boston Scientific designed to prevent stone migration [10]. Many devices have been developed, so that they can be placed proximal to the stone, and serve the dual purpose of collecting stone fragments and preventing proximal migration [11]. As an alternative option, a basket device can be used to encapsulate the stone prior to lithotripsy to catch any migrating fragments [11]. There has also been the development of a water-soluble polymeric gel, BackStop^®^, again by Boston Scientific. It possesses a property called reverse thermosensitivity, where the viscosity of the gel increases at body temperature. This allows it to form a plug beyond the stone, and when irrigated with cold saline solution, it liquidates and can be flushed from the ureter [10]. Holmium YAG laser (Ho:YAG) is the most commonly used laser for stone fragmentation and seems to have significantly less retropulsion, is associated with fewer complications, and can be used for stones in the ureter or kidney, with the use of both rigid and flexible URS [5]. The use of laser safety glasses is a requirement for all OR staff.

Routine pre-operative stenting is not necessary before URS, though it has a role in patients with narrow ureters providing passive dilatation, so that a delayed procedure can then be performed. Similarly, post-operative stenting might be associated with morbidity and should only be used in patients who are at an increased risk of complications, such as previous iatrogenic trauma, impacted ureteric calculi, ureteric perforation, and in conditions such as solitary kidneys, pregnancy, and a history of retroperitoneal fibrosis [5, 7, 11]. Although the ideal duration of post-operative double J stent is not known, most urologists favour its use for 1–2 weeks after URS [5]. It should be placed with the guide wire held taut, and its position should be checked both cystoscopically and fluoroscopically [6].

## Technique of ureteroscopy

Initially, a cystoscopy should be performed to visualise the bladder, exclude any gross malignancy, and to identify the ureteric orifice (UO). At this point, a safety guidewire can be placed via the UO in the kidney, to allow repeated atraumatic access to the ureter/kidney. EAU guidelines advocate the use of a safety guide wire [5], though it is possible to perform the procedure without using it. Access to the UO is usually straight forward, and care must be taken with the cystoscope at the bladder neck, especially in men with an enlarged vascular prostate gland [13]. Tips for difficult access include using the ureteroscope itself to insert the wire under direct vision, by placing its tip at the UO [13].

Once satisfactory guidewire access is achieved, a ureteral catheter can be inserted over the wire allowing the endourologist to perform a retrograde ureteropyelography with fluoroscopic dye. This catheter is typically a 5–6 F with an open tip, and can be used as a conduit to inject dye or to obtain urine samples for cytology [6]. If pus is drained from the ureter at this point, then one should simply place a stent to drain the infection and delay any intervention [7]. The ureteric catheter must be primed prior to use to avoid injecting air into the renal system. Fluoroscopy helps to delineate the anatomy; however, in patients with image-confirmed distal stones, distal URS can be performed prior to this to prevent potential stone migration [2]. Once a safe passage has been visualised, the guidewire can be advanced into the renal pelvis under radiological guidance. It is recommended to use a “flash” of radiation, as opposed to constant screening, to reduce its dose [7]. Once the guidewire is safely inserted in the renal pelvis, if desired, an assistant can ‘pin’ the guide wire to a fixed sterile point to avoid it kinking and to ensure that it does not accidentally get pulled out during any intervention [14]. At this stage, the operating surgeon may wish to change the initial guide wire if it is hydrophilic coated, as they have a tendency to slip out of the urinary tract [6]. It should be changed via an open-ended catheter over the hydrophilic wire as this maintains access to the renal pelvis [6]. In certain cases, there may be difficulty advancing the guide wire due to anatomical barriers. Examples include J-hooking of the distal ureter, or a pathological process such as an impacted stone, tight stricture, or tumour [6]. Techniques to combat this include the additional injection of dye at the level of the obstruction to better define any plausible routes of advance. Alternatively, the length of the ‘floppy tip’ of the guidewire can be increased to provide greater flexibility at the level of obstruction [6]. Difficulty inserting the ureteroscope itself can be combatted with various techniques such as balloon and plastic dilators if necessary [5]. If ureteral access is not possible, insertion of a JJ stent followed by URS after 7–14 days offers an alternative procedure [5], as this helps to gradually dilate the ureter, so a scope can be manoeuvred through it.

## Scope insertion

Before inserting the scope, the bladder should be emptied to avoid compression of the ureteric orifice and facilitate scope advancement [7]. The scope should be held in the dominant hand of the endourologist with the other hand stabilising the scope at the urethral meatus [14]. Care should be taken at all times to keep the ureteroscope straight and to avoid any unnecessary stress on its shaft. If it is difficult to enter the UO, the scope can be rotated 90°–180° which can compensate for the curved beak at its tip [7]. The initial safety guidewire will keep the UO open, and a second ‘navigating’ wire, usually PTFE, can be added to widen access for the scope. It is often easier to follow this navigating wire up the ureter, with the scope between the two wires, enabling any obstacles to be bypassed [7]. If the ureter is snug, and the scope is not easily advancing, it is imperative not to force the scope as there is a high risk of ureteric perforation. When any resistance is felt, the endourologist should re-evaluate the situation; all force exerted on the scope should cease and repeat fluoroscopic dye injection may identify a cause of the obstruction [14]. Hydrophilic wires are more likely to glide past an obstruction than PTFE ones, and if difficulty persists, then a wire with an angled tip could be trialled [7]. If contrast goes beyond the obstruction, then an attempt to pass a hydrophilic wire through the ureteral catheter is indicated [7]. For impacted stones that are hindering any advancement of the procedure, displacement can be attempted either with gentle nudges with the ureteric catheter or scope itself (Billiard Cue technique) or via the scope after fragmenting the stone [7]; however, a novice should not undertake these. Any evidence of iatrogenic mucosal damage is an indication to delay endoscopic intervention and it is then recommended to simply insert a stent over the safety wire [7].

## Indications for semi-rigid ureteroscopy

With rapidly advancing technology, there is a broadening scope for endoscopic urological procedures. As ureteroscopy has both diagnostic and therapeutic roles, it can be used safely to manage a range of conditions including stone disease, ureteral strictures, and ureteric tumours [4, 5]. A ureteroscope of ≤ 8 F is considered safe, and the constant improvement of scopes with narrower lumens enables more patients to be managed endoscopically [5].

## Management of stones

Compared to lithotripsy (SWL), the rapid development of ureteroscopes has allowed management of most ureteric stones, especially for those that need a pre-SWL stent insertion (> 1.5 cm) or are too large (> 2 cm) for SWL [5, 14]. Semi-rigid ureteroscopes can be used selectively for stones in the renal pelvis if the view and approach to the stone looks favourable; otherwise, for stones in the pelvicalyceal system, a flexible URS generally needs to be used [5, 10, 13]. It has also enabled stone management in the obese patient category, who were previous unsuitable for SWL due to the high stone to skin distance [10].

Prior to commencing the procedure, the recommended stone fragmentation device, and other disposals such as guidewire, baskets, and stents should be readily available. The equipment should be selected appropriately: laser settings should be altered according to the stone’s characteristics and the correct size basket selected, with consideration that larger sized shafts will reduce the flow of irrigation fluid and might affect the surgeon’s view [7, 11]. There is no confirmed superior technique of laser fragmentation and the choice of reducing the stone to dust, or to fragments that are easily retrieved is dependent on the surgeon’s preference [11]. One rule which universally applies is that the stone targeted must be visualised at all times, and the surgeon should never attempt ‘blind basketing’ in an attempt to retrieve it or try to pull the basket if it appears to be stuck [7, 10].

In a global study of all ureteroscopy patients by the Clinical Research Office of the Endourological Society (CROES), it was found that the majority of procedures performed for stone disease used semi-rigid ureteroscopes [15]. Semi-rigid URS typically achieves a 95% success rate of stone clearance in distal ureteric calculi [16], as these stones are easier to access with a rigid scope and these fragments are less likely to migrate proximally [17]. Another advantage of the semi-rigid scope is its wider working channel, which allows for improved flow and slightly higher irrigation pressures leading to better vision during the procedure [16]. However, the drawback of semi-rigid URS is its small rate of failure to access, meaning that further procedures are required at a later date [15]. This inadvertently increases the overall cost of the intervention. There is also a potential risk of scope fracture, which is particularly relevant in male patients with a long distance between a relatively rigid prostatic urethra and over more developed psoas muscles to the renal pelvis [17]. Flexible ureteroscopy predominantly has a role in proximal ureteric calculi, and the CROES group reported that it has a marginally higher success rate in stone clearance (85.5%) compared to semi-rigid URS (83.8%) [15]. They are less efficient in distal stones as it is difficult to maintain access to the ureter with the flexible scope [18]. Overall, the CROES study found that there was no significant difference in complication rates between flexible and semi-rigid URS [15]; therefore, the surgeon should weigh the choice of scope with stone location, availability, cost, and personal preference (Table 1).

**Table 1:** Summary of the ‘tips and tricks’ necessary for difficulties during semi-rigid URS

	Step 1	Step 2	Step 3	Step 4	Step 5
Difficulty at the Bladder neck/UO [7]	Be cautious with the scope at the bladder neck to avoid any injury (e.g., enlarged prostate)	Rotate the scope 90°–180° at the UO to compensate for the scope’s curved beak	Use a hydrophilic tipped wire—this can load the ureteric catheter to guide it in if struggling, and approach infero-laterally	Can use fluoroscopy to define the anatomy and identify any ‘fish hooking’ of the lower ureter	Can insert the ureteroscope itself into the UO and use this to insert the guidewire
Difficult within the ureter [5–7]	Place an additional navigating wire to open up the UO/ureter and allow passage of the scope	Increase the length of the ‘floppy’ tip of the wire or use an angled tip (J-tip) wire to negotiate the ureter	An additional injection of fluoroscopic dye at the level of the obstruction can help identify a route	Use of balloon and plastic dilators to gradually stretch the ureter to enable advancement	If unable to advance the scope, place a JJ stent and delay the intervention
Stuck basket [7, 10, 19]	Avoid any forceful or blind intervention with the basket (do not pull)	Fragmentation of the grasped stone may be required to freely remove the basket	If unable to dislodge the basket after stone fragmentation, consider cutting the basket wires to free it		
Impassable stone [7]	Should only be attempted by a competent Endourologist (not a novice procedure)	Gentle nudging with the ureteric catheter in attempt to dislodge the stone	Similarly, the ureteroscope itself can be used to shift the stone (Billiard Cue technique)	If unsuccessful, fragmentation of the stone under vision and then the scope can be advanced	If the stone causes ‘Z’ configuration of the upper ureter, use sequential advances of the wire to navigate the bends
Poor views [11]	Ensure the scope is focused, brightness of light is adjusted and the white balance is complete	Use smaller ancillary equipment as the narrower shafts will occlude the irrigant flow less	Consider increasing irrigation pressures if any bleeding occurs to improve views	If still unsuccessful, place a JJ stent and delay the intervention	

*UO* ureteric orifice

## Ureteric strictures

A ureteric stricture is defined as a narrowing of the ureter, which causes a functional and/or anatomical obstruction [13]. They can be congenital, idiopathic, or acquired, and ureteroscopy is both a recognised iatrogenic cause and a treatment option for it. The gold standard approach to ureteral stricture repair was open surgery [13]; however, with advancing ureteroscopic techniques, they are rapidly overtaking the more invasive approach. Open management of ureteral strictures is dependent on their location; distal strictures often require re-implantation, whereas proximal and mid-ureteric strictures can be managed with a boari flap, ureteroureterostomy, or an ileal transposition [20].

Using the ureteroscope, the options lie between balloon dilatation, sequential endoscopic dilatation or laser endoureterotomy [5, 11, 21]. The success of balloon dilatation ranges from 48 to 82% [13]. The technique is performed by inserting the balloon device over the safety guidewire under fluoroscopic guidance, helping to delineate the anatomy of the stricture. The balloon can then be inflated in a controlled manner to gradually dilate the stenotic area [13]. A post-procedure ureteric stent is then placed after the procedure, the time period of which is dependent on the surgeon’s preference, and can range from 1 to 8 weeks [13]. Laser endoureterotomy provides comparable long-term results to open surgery, with lower morbidity rates and shorter recovery times [22]. Alike to stone disease, the Holmium YAG laser is the most commonly used device for this procedure as it has low complication rates [21]. Initially, the stricture segment needs balloon dilation sufficiently to allow easy passage of a semi-rigid ureteroscope [23]. The scope can then be advanced, over a safety wire, so that the endoureterotomy can be performed under direct vision. The stricture is excised with the laser device, and adequate depth of the incision can be confirmed by visualisation of the extraureteric fat and extravasation of contrast radiographically [23]. A stent is recommended post-operatively as it promotes ureteric healing, prevents extravasation of urine, and reduces the incidence of re-stricturing [13]. The contraindications to ureteroscopic treatment are strictures longer than 1.5 cm, poor renal function (eGFR < 25)m and severe dilatation of the renal pelvis [13].

## Urothelial pathology: a diagnostic and therapeutic role of URS

Ureterorenoscopy allows direct visualisation of any pathology in the lumen of the ureter or the kidney. When URS is used for diagnostic purposes, additional care must be taken to minimise any scope or guidewire associated trauma as damage to the delicate mucosal layers could be mistaken for a pathological process [14]. URS has added benefits of being a diagnostic tool and urine samples can be taken for cytology (preferably pre-instrumentation) with tissue samples for histology using the scope itself [11]. It is a fairly non-invasive means of investigating significant urinary tract pathology. There are two methods of urothelial tumour biopsy: cold-cut technique using a stone basket or single use biopsy forceps [11]. Care should be taken when avulsing the biopsy to avoid perforation of the ureter. The development of ureteroscopy as a diagnostic tool has also fuelled the development of ancillary equipment, such as the BIGopsy^®^ device by Cook Medical, which allows the largest possible tissue sample to be taken, on removal of the scope [10]. Though the standard treatment for urothelial tumours is nephroureterectomy (NU), it is not suitable for all patient groups, such as those with a solitary kidney, chronic kidney disease, bilateral malignant disease, or patient preference to avoid NU [6, 24]. The available treatment options using the ureteroscope include using the cold-cut technique to debulk the tumour or vaporisation with the Holmium YAG laser [6].

## Specialist cases for URS: urolithiasis in pregnancy, obese, paediatric, and bleeding diathesis

Management of stone disease in certain patient groups such as in pregnancy, obesity, paediatrics, and patients with bleeding diathesis is challenging and poses a management dilemma [25–29]. While SWL is contraindicated in pregnancy and patients with bleeding diathesis, it is less effective in obese and needs a GA in paediatric patients. Ureteroscopic approach can be used with a relatively good success rate in these patient groups and a rigid URS has a good success rate for majority of ureteric stones in these high-risk patients [25–29].

## Conclusion

The role of semi-rigid ureteroscopy has evolved over the last 3 decades but is still the mainstay of management in diagnostic and therapeutic interventions in the ureter.
